# Furosemide-induced severe hypokalemia with rhabdomyolysis without cardiac arrest

**DOI:** 10.1186/1472-6874-13-30

**Published:** 2013-07-09

**Authors:** Wolfgang Ruisz, Claudia Stöllberger, Josef Finsterer, Franz Weidinger

**Affiliations:** 1 Krankenanstalt Rudolfstiftung, Juchgasse 25, A-1030 Wien, Österreich; 2 Steingasse 31/18, A-1030 Wien, Österreich

**Keywords:** Furosemide, Hypokalemia, Rhabdomyolysis

## Abstract

**Background:**

Hypokalemia induced by diuretic abuse is a life-threatening emergency.

**Case presentation:**

A 22-years-old female nurse with a body mass index 18 suffered from myalgias, vomiting and diarrhea. Blood tests revealed hypokalemia with a lowest value of 1.1 mmol/l, moderate hyponatremia, metabolic alkalosis, mild renal insufficiency and creatinphosphokinase-elevation. Since hypokalemia and alkalosis were unexplained, she was asked for diuretic-intake. She confessed that she has taken 250 mg furosemide/day for the last 4 months to improve the shape of her muscles. Furosemide tablets were given to her by a physician attending the gym where she exercised. After electrolyte substitution, laboratory abnormalities regressed and no cardiac arrests were observed. Psychiatric investigation diagnosed an adjustment disorder.

**Conclusion:**

Furosemide abuse has to be considered even in underweight individuals, especially if they have a psychiatric instability or work in health care institutions.

## Background

Chronic abuse of furosemide as cause of hypokalemia has been repeatedly reported [1-5]. Extreme furosemide-induced hypokalemia with a serum potassium level of 1.1 mmol/l, is rare and associated with life-threatening cardiac arrest and rhabdomyolysis [6-8]. Typically, furosemide-induced hypokalemia is characterized by hypernatremia, hypocalcemia, hypomagnesemia, high urine calcium creatinine ratio, high urine sodium excretion and high urine calcium/creatinine ration. Furosemide-induced hypokalemia may also present without these electrolyte abnormalities and only be detected by the history, as shown by the following case.

## Case presentation

**Figure 1:**
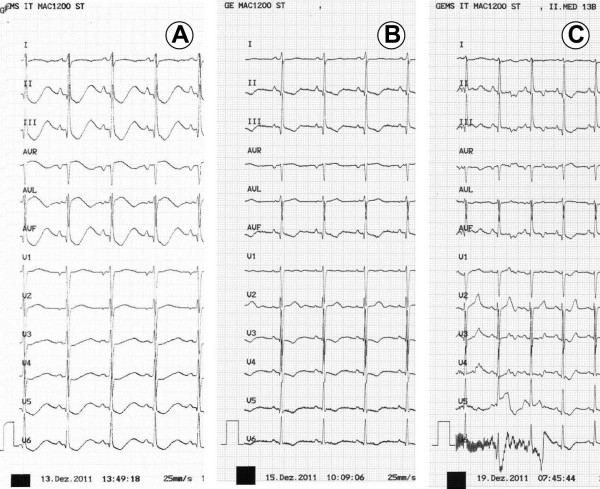
**Electrocardiographic recordings on day 1 (A), day 3 (B) and day 7 (C) showing ST abnormalities and QT prolongation on days 1 and 3 which normalized on day 7.** The QTc according to Bazett’s formula was 670 msec (day 1), 405 msec (day 3) and 401 msec (day 7).

A 22-years-old female nurse was admitted because of myalgias since 10 days, vomiting and diarrhea since 4 days, and generalized weakness and dizziness since 3 days. She had a history of Raynaud’s disease and of a restless leg syndrome since 9 years. She reported to take no medication against these disorders. The family history was negative for renal and muscle disease. Clinical examination showed an alert young female with 160 cm length and 46 kg weight (Body mass index 18). Blood pressure was 115/80 mm Hg and the respiratory rate 12/min. No abnormalities were found at clinical examination. There were no abnormalities in the skin turgor. Blood tests revealed severe hypokalemia with a lowest value of 1.1 mmol/l, moderate hyponatremia, metabolic alkalosis, mild renal insufficiency and creatinphosphokinase (CK) elevation (Table 1). Urine electrolytes on day 2 showed a decreased excretion of potassium (9 mmol/l, normal range 20–80 mmol/l) and sodium (29 mmol/l, normal range 54–150 mmol/l). Thyroid function tests were normal. The electrocardiogram showed ST-segment abnormalities and a prolonged QT interval (Figure 1). Since the severe hypokalemia and alkalosis were not explained by the gastrointestinal problems, she was asked for intake of diuretics even 12 hours after admission. She confessed that she has taken 250 mg furosemide/day for the last 4 months in order to improve the shape of her muscles and to have a more brawny appearance. She had received the tablets from a physician who attended the same gym where she exercised. The patient was diagnosed as suffering from furosemide-induced hypokalemia and rhabdomyolysis. Parenteral and enteral substitution of potassium, sodium and magnesium was started. The serum electrolyte levels normalized within 4 days. CK levels gradually decreased, normalized after two weeks and the myalgias regressed. A psychiatric investigation excluded suicidality, depression or eating disorder, diagnosed an adjustment disorder and recommended psychotherapy. Her heart rhythm was monitored during 7 days and did not show any arrhythmias. Echocardiography did not disclose any cardiac abnormalities, and the electrocardiogram normalized (Figure 1). After 7 days she left the hospital and returned for blood tests after one week.

**Table 1:** Laboratory findings

**Parameter**	**Reference value**	**Day 1**	**Day 1**	**Day 2**	**Day 2**	**Day 3**	**Day 3**	**Day 5**	**Day 7**	**Day 8**	**Day 15**
		**12:02**	**22:58**	**05:47**	**21.38**	**07:42**	**18:10**	**09:45**	**10:42**	**08:08**	**12:21**
BUN	9.0-20.0 mg/dl	25	21	21	NM	12	9	4	6	8	14
Creatinine	−1.1 mg/dl	1.64	1.15	NM	NM	0.87	1.00	0.74	0.80	0.84	0.81
GFR	>90 ml/min/1.73 m	42	63	NM	NM	87	74	104	95	90	94
Sodium	136.0-145.0 mmol/l	130	129	132	132	138	136	137	140	140	139
Potassium	3.3-5.1 mmol/l	1.1	1.6	1.5	1.4	1.7	2.8	4.5	5.4	4.9	5.0
Chloride	98.0-106.0 mmol/l	90	92	97	98	101	97	100	NM	NM	99
Calcium	2.1-2.7 mmol/l	2.66	2.0	2.0	2.0	2.1	2.0	2.1	NM	NM	2.8
pH	7.33-7.42	7.549	7.514	7.468	7.454	7.357	7.453	7.467	NM	NM	7.371
HCO_3_^-^	22-26 mmol/l	31.4	28.6	29.0	28.4	25.3	30.9	33.3	NM	NM	29.0
P_CO2_	38-42 mmHg	36.1	34.2	40.9	42.3	52.1	48.0	35.7	NM	NM	57.0
CK	−144.0 U/l	15966	13143	11570	NM	9882	12043	5703	1867	1070	102
Leukocytes	4.0-9.0/nl	18.1	NM	NM	NM	9.5	8.9	NM	NM	5.5	5.9
Erythrocytes	4.0-5.2/pl	5.19	NM	NM	NM	3.28	3.48	NM	NM	3.54	3.55
Hemoglobin	12.0-16.0 g/dl	16.6	NM	NM	NM	10.4	11.2	NM	NM	11.4	12.1
Hematocrit	38.0-48.0%	42.7	NM	NM	NM	27.8	29.6	NM	NM	35.0	35.2

*GFR* = glomerular filtration rate, estimated according the Modified Diet in Renal Disease formula; *CK* = Creatinphosphokinase.

## Discussion

This patient with severe hypokalemia is interesting for the following reasons: It is extremely rare that serum potassium levels decrease below 2 mmol/l and that such low levels are tolerated without cardiac arrests. Previous reports show that severe hypokalemia is associated with cardiac arrests and the necessity for cardiopulmonary resuscitation [6]. That our patient did not develop any arrhythmia despite severe hypokalemia may be explained by her healthy heart as demonstrated by the normalization of electrocardiography and normal echocardiographic findings, by the chronic development of hypokalemia and, possibly, by unknown genetic protective factors [9].

Furosemide-induced hypokalemia, also termed “Pseudo-Bartter-Syndrome”, is found mainly in young females who work in health-care institutions, like our patient, who may have an easy opportunity to watch the impressive effect of diuretics and have an easier access to the drugs than people who work outside health-care institutions [1-5]. Motivation for surreptitious furosemide intake is the intention to slim, to avoid edema or, like in our patient, to change the shape of the body. Furosemide may be taken also unknowingly as an undeclared component of “health teas” and lead to hypokalemia [10]. Previous reports indicate that extensive diagnostic measures may be necessary to establish the diagnosis, thus our experience to confront the patient early in the diagnostic process with the suspicion of diuretic-intake seems reasonable.

Neurologic manifestations of hypokalemia comprise paralysis and rhabdomyolysis [11-13]. Rhabdomyolysis in hypokalemia either remains asymptomatic or manifests clinically with muscle pain, cramps and weakness. The reason for rhabdomyolysis in hypokalemia is assumed to be muscle ischemia. Physiologically elevated potassium, for example due to exercise, causes vasodilatation and thus increases the muscular blood flow [14]. In case of hypokalemia, increase of serum potassium is hindered resulting in relative ischemia, muscle cramps, and in case of severe depletion, muscle necrosis and rhabdomyolysis [14]. Another pathomechanism may be impaired myocyte metabolism or membrane dysfunction, resulting in muscle-cell breakdown or membrane leakage [15].

It remains unclear why our patient did not develop the electrolyte abnormalities typical for furosemide-induced hypokalemia. Her concomitant hyponatremia cannot be explained by furosemide-intake since furosemide may lead to hypernatremia. Probably, other so far unknown factors may have contributed to hyponatremia. Unfortunately, no urine chloride levels have been measured. One would expect them to be elevated, which could have been helpful in the patient’s workup if she had not admitted diuretic intake.

Additionally it remains unknown whether she had a predisposition to hypokalemia or rhabdomyolysis. Disorders which may lead to hypokalemia like Gitelman syndrome, Bartter syndrome, Andersen-Tawil syndrome, renal tubular acidosis or periodic hypokalemic paralysis were excluded by the negative family history, the absence of a periodic occurrence and the absence of hypomagnesemia, hypocalcemia and acidosis [16,17].

## Conclusions

This case shows that furosemide-induced severe hypokalemia may present without cardiac arrest, may be survived without sequelae and may be accompanied by transient rhabdomyolysis. Chronic furosemide abuse in order to lose weight has to be considered even in underweight individuals, especially if they have a psychiatric instability or work in health care institutions.

## Consent

“Written informed consent was obtained from the patient for publication of this Case report and any accompanying images. A copy of the written consent is available for review by the Series Editor of this journal.”

## Competing interests

The authors declare that they have no competing interests

## Authors’ contributions

WR investigation of the patient. CS literature research, drafting of the manuscript, corresponding author. JF literature research, drafting of the manuscript. FW drafting of the manuscript. All authors read and approved the final manuscript.

## Pre-publication history

The pre-publication history for this paper can be accessed here:

http://www.biomedcentral.com/1472-6874/13/30/prepub
